# Ebola Virus Disease Outbreak in Isiro, Democratic Republic of the Congo, 2012: Signs and Symptoms, Management and Outcomes

**DOI:** 10.1371/journal.pone.0129333

**Published:** 2015-06-24

**Authors:** Thomas Kratz, Paul Roddy, Antoine Tshomba Oloma, Benjamin Jeffs, Diana Pou Ciruelo, Olimpia de la Rosa, Matthias Borchert

**Affiliations:** 1 Institute of Tropical Medicine and International Health, Charité-Universitätsmedizin, Berlin, Germany; 2 Médecins Sans Frontières—Spain, Barcelona, Catalonia, Spain; 3 Independent Consultant Epidemiologist, Barcelona, Catalonia, Spain; 4 University of Kisangani, Orientale Province, Kisangani, Democratic Republic of the Congo; 5 Bradford Teaching Hospitals Foundation Trust, Bradford, West Yorkshire, United Kingdom; 6 Médecins Sans Frontières—Belgium, Brussels, Belgium; 7 Drassanes Tropical Medicine and International Health Centre, Barcelona, Catalonia, Spain; Division of Clinical Research, UNITED STATES

## Abstract

Data collected during the 2012 Ebola virus disease (EVD) epidemic in the Democratic Republic of the Congo were analysed for clinical signs, symptoms and case fatality of EVD caused by Bundibugyo virus (BDBV), establishment of differential diagnoses, description of medical treatment and evaluation of the quality of clinical documentation. In a quantitative observational prospective study, global epidemiological data from 52 patients (34 patients within the community, 18 patients treated in the Ebola Treatment Centre) were entered anonymously into a database, subsequently matched and analysed. Relevant findings include an over-representation of females among community EVD cases (85.3%) and of community EVD cases in the age group of 15-54 years (82.4%). All ETC patients had fever (55.6% of all 18 ETC patients during their hospital stay) or self-reported fever (88.2% upon admission) at some point of time during their illness. Major symptoms of ETC patients during hospital stay included asthenia (82.4%), anorexia (82.4%), myalgia (70.6%), sore throat/difficulty swallowing (70.6%), arthralgia (76.5%) and nausea (70.6%). Gastrointestinal signs and symptoms (nausea, diarrhoea, vomiting) (76.4%) as well as general pain (94.1%) were frequent in ETC patients. The median duration of EVD was 18 days, while the mean incubation period was 11.3 days. Differential diagnosis of EVD included malaria (28.3%), intestinal parasitosis (10.9%), and infectious syndrome (10.9%). There was also an important variation in clinical evolvement. Quality of documentation was adversely affected by the way patient file contents were transferred from inside to outside the high-risk zone, entailing a mean mismatch value of 27.3% between patient file contents inside vs. outside the high-risk zone. This study adds further description of EVD (frequently non-specific signs and symptoms, non frequent bleeding, a long incubation period, long duration of disease) and emphasizes the need for improving clinical monitoring and documentation in EVD outbreak settings.

## Introduction

Ebolavirus disease (EVD) is an acute severe disease with a high case fatality risk (CFR). Four ebolaviruses have been identified prior to 2007: Ebolavirus (EBOV), Sudan virus (SUDV), Taï Forest virus (TAFV) and Reston virus (RESTV). EBOV and SUDV are associated with large outbreaks in Equatorial and Western Africa with high CFRs [1–3].

In 2007, a newly identified ebolavirus, Bundibugyo virus (BDBV), was discovered during an outbreak in Uganda [4, 5]. The epidemic yielded 56 laboratory-confirmed cases. Out of 43 cases ä, 17 were fatal (CFR 40%) [4]. The most frequent sign was non–bloody diarrhoea (81%), the most frequent symptom asthenia (77%), while only seven patients (27%) showed haemorrhagic signs [5]. No further outbreak of EVD caused by BDBV was observed until 2012. [6–9]

### The 2012 Ebola virus disease outbreak

The medical humanitarian organisation Médecins Sans Frontières (MSF) received notice on 2 August 2012 that four patients in Isiro town, Haut-Uele District, Province Orientale, Democratic Republic of the Congo (DRC) had died of an unknown cause with unexplained bleeding [Carolina Nanclares, MSF, personal communication]. Blood samples of two patients were subsequently sent to the Uganda Virus Research Institute in Entebbe, Uganda [10]. On 9 August an MSF team arrived in the field to set up a provisional Ebola Treatment Center (ETC) within the compound of the General Hospital in Isiro, which was declared functional on 12 August. On 16 August, BDBV was confirmed in the above mentioned two patients, and the epidemic of EVD was declared on 17 August by the Congolese Ministry of Health (MoH) [11]. On 21 August, the ETC structure was expanded, and additional human and material resources were made available. Other outbreak response contributors included the Institut National de la Recherche Biomédicale (Kinshasa DRC), the World Health Organisation (WHO), Centers for Disease Control and Prevention (CDC, Atlanta, GA), the Public Health Agency of Canada (PHAC), UNICEF and the Congolese Red Cross [10].

Clinical management of EVD patients followed the MSF 2008 Filovirus Haemorrhagic Fever Guidelines [12]. Suspect cases were defined as any case with fever and at least three signs and/or symptoms compatible with EVD from a predefined list, or any unexplained death. Probable cases were suspect cases with prior contact with a probable or confirmed EVD case. Confirmed cases were defined as Polymerase Chain Reaction (PCR) or Immunoglobulin M (IgM) ELISA positive [10]. For details see the MoH case definition in the additional online material. Patients admitted to the ETC for diagnosis and treatment were self-referrals, or referrals from other hospitals or from surveillance teams working in the community. Laboratory confirmation through PCR and IgM-ELISA was conducted on-site by CDC and PHAC; results were available 4 to 24 hours after receipt of sample. On 26 Nov 2012 MoH declared the end of the epidemic with 36 confirmed, 21 probable and 5 suspect cases, including 34 fatalities (CFR 54,8%) [11]. All cases originated from Isiro Health Zone.

## Rationale and Objectives

Demographic and clinical data on EVD caused by BDBV, e.g. on signs, symptoms, treatment and case fatality are sparse. The epidemic in 2012 was only the second ever observed, and the investigation of the first BDBV outbreak in 2007 was hampered by the limited quality of clinical documentation, including data not or only haphazardly recorded, or clinical records destroyed for biosafety concerns [5]. From the 2012 EVD outbreak caused by BDBV, detailed clinical data were available for 31 patients.

For some patients, an initial diagnosis other than EVD was established, and EVD was only diagnosed later. Thus, as opposed to Roddy et al [5], differential diagnoses of EVD could be established.

As clinical documentation has previously been shown to be poor in outbreaks due to filoviruses [5, 13], we evaluated the quality of the documentation. Solid clinical documentation is a pre-requisite for evaluating innovative case management approaches, such as antiviral drugs and convalescent blood or plasma, currently investigated in West Africa [14].

The general objective of our study was therefore to depict the clinical features, medical treatment and outcome of EVD during the 2012 outbreak in Isiro. Specific objectives were to: (i) Analyse the demographics of patients with EVD, (ii) Establish differential diagnoses, (iii) Analyse the distribution of clinical signs and symptoms of suspected EVD prior to its laboratory confirmation, (iv) Analyse the distribution of clinical signs and symptoms of EVD once laboratory confirmed, (v) Establish the case fatality risk, (vi) Describe the monitoring of patients, (vii) Assess the medical treatment provided, and (vii) Evaluate the quality of clinical documentation.

## Setting and Methods

**Fig 1:**
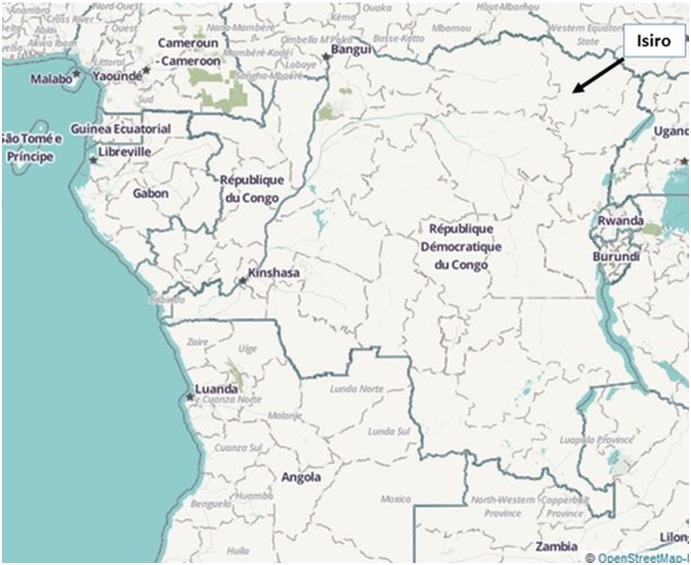
Map of the Democratic Republic of the Congo. Isiro, capital of the Haut-Uele District, is situated in north-eastern DRC.

Isiro, the capital of Haut-Uele District, is situated between the equatorial forest and the savannah in the north-eastern part of DRC. The primary spoken languages are Lingala and, less frequently, Swahili. The town and its greater urban area comprise approximately 225,000 inhabitants and 71 health facilities, including 37 situated in the Isiro urban area. [10, 15] (Fig 1)

**Fig 2:**
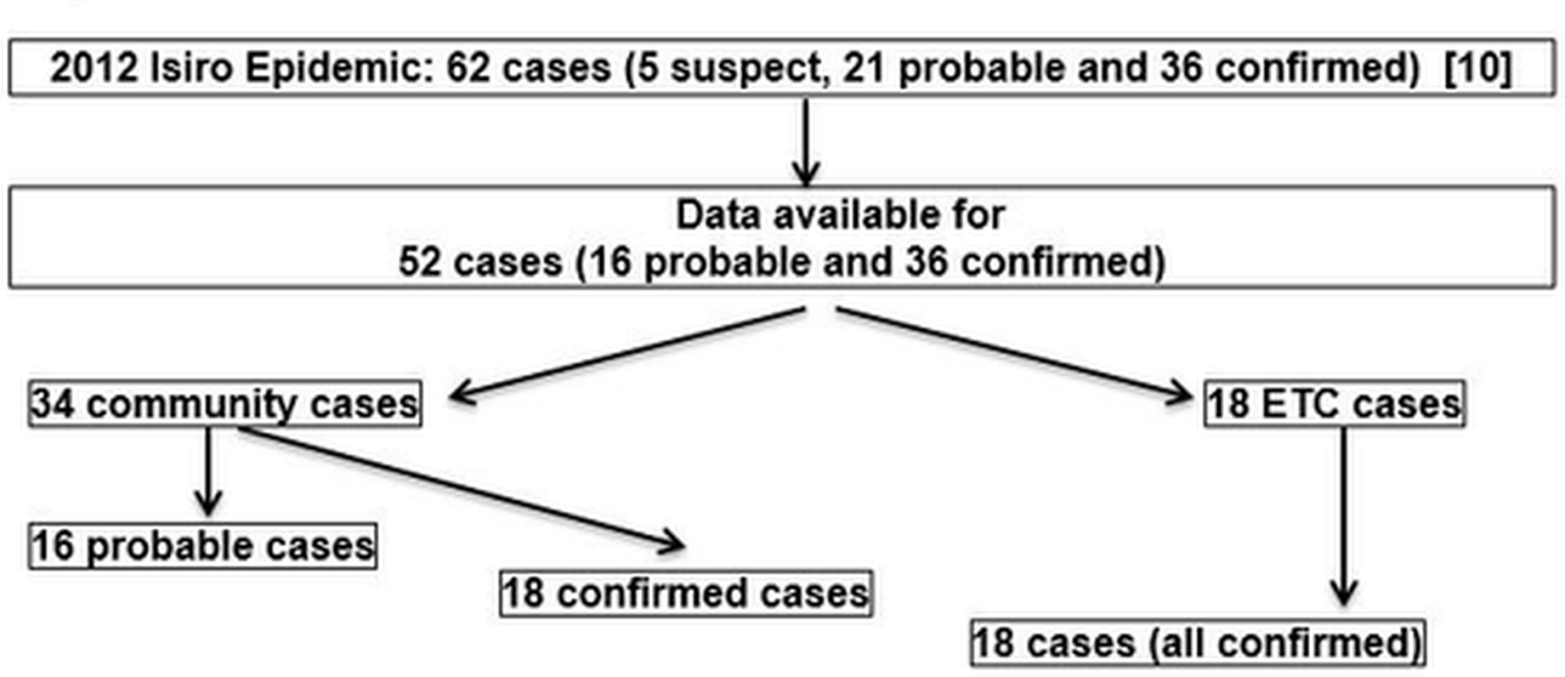
Case numbers of the 2012 Isiro Epidemic. The Isiro Epidemic yielded 62 cases (5 suspect, 21 probable and 36 confirmed). Clinical documentation of 52 cases (16 probable, 36 confirmed) could be retrieved and analysed. Out of these, there were 16 probable and 18 confirmed community cases, which never reached the Ebola Treatment Centre (ETC). The 18 patients who reached the ETC were all confirmed cases.

This study was quantitative and observational. We used demographic and clinical data routinely collected by MSF staff for 52 EVD cases (probable n = 16; confirmed n = 36) identified during the 2012 EVD epidemic (Fig 2).

Community cases were defined as patients who never reached the ETC. Probable community cases included those who died and had been buried, preventing further laboratory testing. Confirmed community cases included patients who survived without hospitalisation, for which BEVD was diagnosed retrospectively using serology. Confirmed cases included 14 survivors and 22 deaths. Four of the fatal cases died before admission to the ETC; infection with BDBV was detected *post mortem* by IgM ELISA or PCR.

Clinical data from 18 laboratory-confirmed EVD patients, who were patients on the ETC in Isiro, were anonymised and double entered in EpiData 3.1 (EpiData Association, Odense, Denmark). Files of the ETC inpatients included a case notification chart with signs and symptoms upon notification as suspect case, a follow–up chart documenting the presence or absence of 31 signs and symptoms on a daily basis during hospital stay, and a temperature chart documenting body temperature and administered treatments. Fever was defined as an axillary body temperature of ≥37.5°C. Haemorrhage was defined as any haemorrhagic sign, excluding “red eyes” that may have been due to conjunctivitis and not to haemorrhage [4]. Gastrointestinal complaints with a potential for dehydration were defined as nausea or vomiting (not distinguishable in the patient files) or diarrhoea. Pain was defined as headache, myalgia, arthralgia, back pain, chest pain, abdominal pain, sore throat and retro-orbital pain. For survivors, duration of disease was defined from symptom onset until the last recording of a symptom prior to discharge. The incubation period was calculated when a single contact lasted one day only and the date of symptom onset was recorded. Doctor’s notes from ward rounds in the ETC were available and provided additional clinical data (n = 18). From health facilities other than the ETC, patient data were available as free text only (n = 13). These data were entered into Microsoft Word forms for analysis. Detailed clinical information was thus available from 31 patients.

Some patients who sought treatment in another health facility without raising suspicion of EVD were sent home and later admitted to the ETC. Other patients were initially diagnosed with another condition in another health facility, and later referred to the ETC as suspect EVD case. For these patients, the differential diagnosis of EVD could thus be established.

In the ETC, there was a low-risk zone designated for e.g. dressing up, storing and handling drugs and other equipment. Healthcare staff dressed up in personal protective equipment (PPE) accessed a high-risk zone (HRZ), where the patients were situated. Data from patient files were routinely transferred from inside to outside the HRZ in the following manner: A clinician established the original patient file consisting of symptoms follow-up chart, temperature and medication chart and free text notes inside the HRZ. After completing the ward round, undressing, and leaving the HRZ the clinician established from memory a copy of the patient file in order to make it accessible outside the HRZ. He or she had thus to remember clinical details of up to six patients treated inside the HRZ at the same time. Comparing the original follow-up charts inside the HRZ with their copies outside the HRZ allowed to determine how reliable this method of data transfer was. Such comparison was possible for 14 patients.

Frequencies, proportions and two-sided Fisher Exact Tests were computed with STATA 12.1. (Stata Corp. College Station, Texas, USA). Microsoft EXCEL 14.1.0 was used for processing external data (e.g. age structure of the Congolese population) and aggregating free-text patient data. TK’s personal observations from his work as a medical practitioner in the ETC were included when relevant. To demonstrate the variability of the clinical course of EVD we document a case series of four patients with particularly detailed clinical records.

Since the study was observational, relied on existing surveillance and clinical data and did not collect additional data for research purposes, the risk for study participants is limited to the disclosure of their identities. Utmost care was taken to secure confidential data processing and storage, all patient identifiers such as name, address and ethnic group were removed prior to data entry and analysis.

Ethics clearance was obtained from the Ethics Review Board (ERB) of Kisangani University/DRC. Individual patient consent for the use of such routine data, including those used in the case series, was not deemed necessary by the ERB of Kisangani University. The MSF ERB issued an exemption note stating that MSF ERB review is not necessary, provided that individual patient identities are not revealed.

## Results

### Demographics

Compared with national demographic data [16] females were significantly overrepresented among EVD cases in the community (50.2% in the general population vs. 85.3% in community cases, p<0.001, vs. 61.1% in ETC patients p = 0.48; Tables 1 and 2).

**Table 1:** Sex and Age distribution of EVD Cases.

		N (%) Community Probable cases	N (%) Community Confirmed cases	N (%) Subtotal Community cases	N (%) *ETC Confirmed Cases	N (%) TOTAL All cases
**Sex**	**Male**	3 (18.8%)	2 (11.1%)	5 (14.7%)	7 (38.9%)	12 (23.1%)
	**Female**	13 (81.3%)	16 (88.9%)	29 (85.3%)	11 (61.1%)	40 (76.9%)
**Age (years)**	**< 5**	1 (6.3%)	1 (5.6%)	2 (5.9%)	1 (5.6%)	3 (5.8%)
	**5–14**	0 (0.0%)	1 (5.6%)	1 (2,9%)	0 (0.0%)	1 (1.9%)
	**15–24**	1 (6.3%)	0 (0,0%)	1 (2.9%)	3 (16.7%)	4 (7.7%)
	**25–34**	4 (25.0%)	3 (16.7%)	7 (20.6%)	5 (27.8%)	12 (23.1%)
	**35–44**	2 (12.5%)	10 (55.6%)	12 (35.3%)	2 (11.1%)	14 (26.9%)
	**45–54**	5 (31.3%)	3 (16.7%)	8 (23.5%)	4 (22.2%)	12 (23.1%)
	**55–64**	3 (18.8%)	0 (0.0%)	3 (8.8%)	2 (11.1%)	5 (9.6%)
	**65–74**	0 (0.0%)	0 (0.0%)	0 (0.0%)	0 (0.0%)	0 (0.0%)
	**≥75**	0 (0.0%)	0 (0.0%)	0 (0.0%)	1 (5.6%)	1 (1.9%)
	**TOTAL**	16	18	34	18	52

*ETC: Ebola Treatment Center

**Table 2:** General Population Distribution of the Democratic Republic of the Congo (DRC).

		N (%) General Population *DRC
**Sex**	**Male**	37,630,328 (49.8%)
	**Female**	37,876,980 (50.2%)
**Age (years)**	**0–14**	32,853,392 (43.5%)
	**15–24**	16,069,730 (21.3%)
	**25–54**	21,959,171 (29.1%)
	**55–64**	2,667,581 (3.5%)
	**≥ 65**	1,957,434 (2.6%)

Source of Table 2: CIA World Factbook 2013

*DRC: Democratic Republic of the Congo

Also overrepresented were individuals in the age group of 15–54 years (50.4% in the general population vs. 82.4% in community cases, p<0.001; vs. 77.8% in ETC patients, p = 0.48), while individuals 14 years or younger were underrepresented (43.5% in the general population vs. 8.8% in community cases, p<0.001; vs. 5.6% in ETC patients, p<0.001).

### Case fatality risk (CFR)

The CFR for community cases was 55.9%, i.e. 93.8% for probable and 22.2% for confirmed cases. CFR of ETC cases (all confirmed) was 50.0%. ETC CFR was insignificantly higher in females (63.6%) than in males (28.6%, p = 0.15; Table 3), and insignificantly higher in the age group of 15–54 years than in all other age groups combined (57.1% vs. 25.0%, p = 0.58; Table 3).

**Table 3:** Case fatality risks (CFR) by sex and age.

		CFR n/N(%) Community Probable cases	CFR n/N(%) Community Confirmed cases	CFR n/N(%) Subtotal Community cases	CFR n/N(%) ETC* Confirmed Cases	CFR n/N(%) TOTAL All cases
**Sex**	**Male**	3/3 (100.0%)	0/2 (0.0%)	3/5 (60.0%)	2/7 (28.6%)	5/12 (41.7%)
	**Female**	12/13 (92.3%)	4/16 (25.0%)	16/29(55.2%)	7/11 (63.6%)	23/40 (57.6%)
**Age (years)**	**< 5**	1/1 (100.0%)	0/1 (0.0%)	1/2 (50.0%)	1/1 (100.0%)	2/3 (66.7%)
	**5–14**	0/0 (0.0%)	0/1 (0.0%)	0/1 (0.0%)	0/0 (0.0%)	0/1 (0.0%)
	**15–24**	1/1 (100.0%)	0/0 (0.0%)	1/1 (100.0%)	2/3 (66.7%)	3/4 (75.0%)
	**25–34**	4/4 (100.0%)	0/3 (0.0%)	4/7 (57.1%)	2/5 (40.0%)	6/12 (50.0%)
	**35–44**	2/2 (100.0%)	3/10 (30.0%)	5/12 (41.7%)	2/2 (100.0%)	7/14 (50.0%)
	**45–54**	5/5 (100.0%)	1/3 (33.3%)	6/8 (75.0%)	2/4 (50.0%)	8/12 (66.7%)
	**55–64**	2/3 (66.7%)	0/0 (0.0%)	2/3 (66.7%)	0/2 (0.0%)	2/5 (40.0%)
	**65–74**	0/0 (0.0%)	0/0 (0.0%)	0/0 (0.0%)	0/0 (0.0%)	0/0 (0.0%)
	**≥ 75**	0/0 (0.0%)	0/0 (0.0%)	0/0 (0.0%)	0/1 (0.0%)	0/1 (0.0%)
	**TOTAL**	15/16 (93.8%)	4/18 (22.2%)	19/34(55.9%)	9/18 (50.0%)	28/52 (53.8%)

ETC*: Ebola Treatment Center

### Differential diagnoses

For 18 out of 36 confirmed EVD cases, written documentation of diagnosis and treatment outside the ETC could be included for analysis. Malaria was the initial diagnosis for 28.3% of these 18 confirmed EVD cases, followed by intestinal parasitosis and infectious syndrome (each 10.9%), gastritis (8.7%), gastroenteritis (6.5%), and dehydration (6.5%) (Table 4).

**Table 4:** Intial diagnoses in patients later confirmed to be EVD cases for n = 18 patients (more than one diagnosis per patient possible).

Differential diagnosis	N (%)
Malaria	13 (28.3%)
Intestinal Parasitosis	5 (10.9%)
Infectious Syndrome	5 (10.9%)
Gastritis	4 (8.7%)
Gastroenteritis	3 (6.5%)
Dehydration	3 (6.5%)
Amebiasis	2 (4.4%)
Salmonellosis	2 (4.4%)
Typhoid Fever	2 (4.4%)
Immunodefiency Syndrome	2 (4.4%)
Dyspeptic Syndrome	1 (2.2%)
Abdominal colics	1 (2.2%)
Cholera	1 (2.2%)
Arterial Hypotension	1 (2.2%)
Hypoglycaemia	1 (2.2%)
TOTAL NUMBER OF DIAGNOSES x PATIENT	46 (100%)

### Body temperature of patients prior to confirmation of EVD

In half of the ETC patients (n = 9 of 18) body temperature had been measured in other hospitals on the day of sign-/symptom onset or later, before EVD was diagnosed at the ETC. All nine patients either self-reported fever (n = 2), or had fever measured (n = 7) at some point in time prior of their ETC stay; five individuals had a fever of 38.5°C or above. During the first day of stay in the ETC, fever was evidenced in three patients only.

### Body temperature evolvement of patients once EVD was confirmed

**Fig 3:**
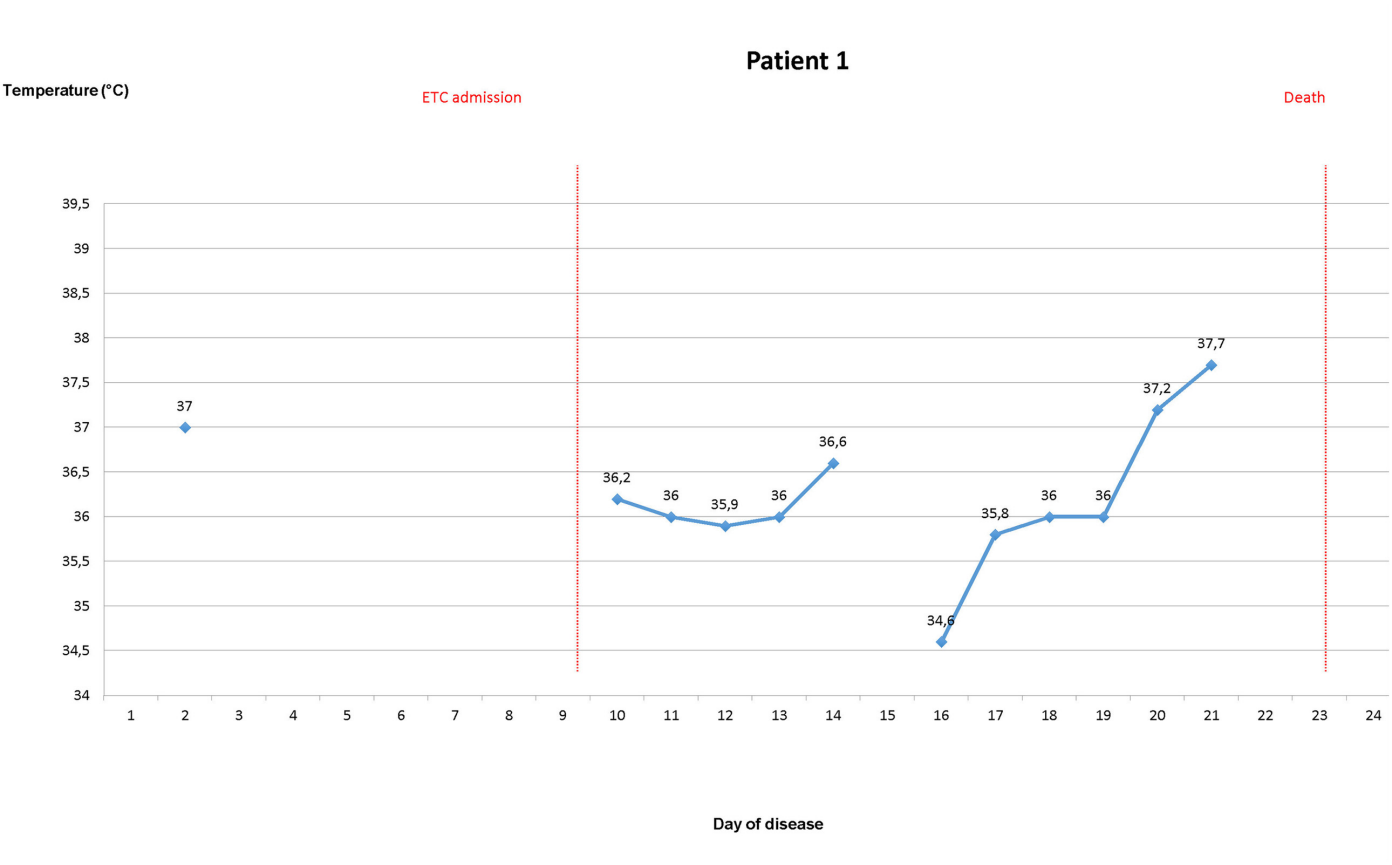
Evolvement of body temperature in patients treated in the Ebola Treatment Centre (ETC). In five inpatients of the ETC body temperature could be followed up from the second or third day of disease onwards. Four of those patients had fever at least on one day. Three patients showed a pattern with fever during the beginning of disease, which lasted no longer than until the 12^th^ day of disease. Three patients had high fever ≥ 38.5°C.

**Fig 4:**
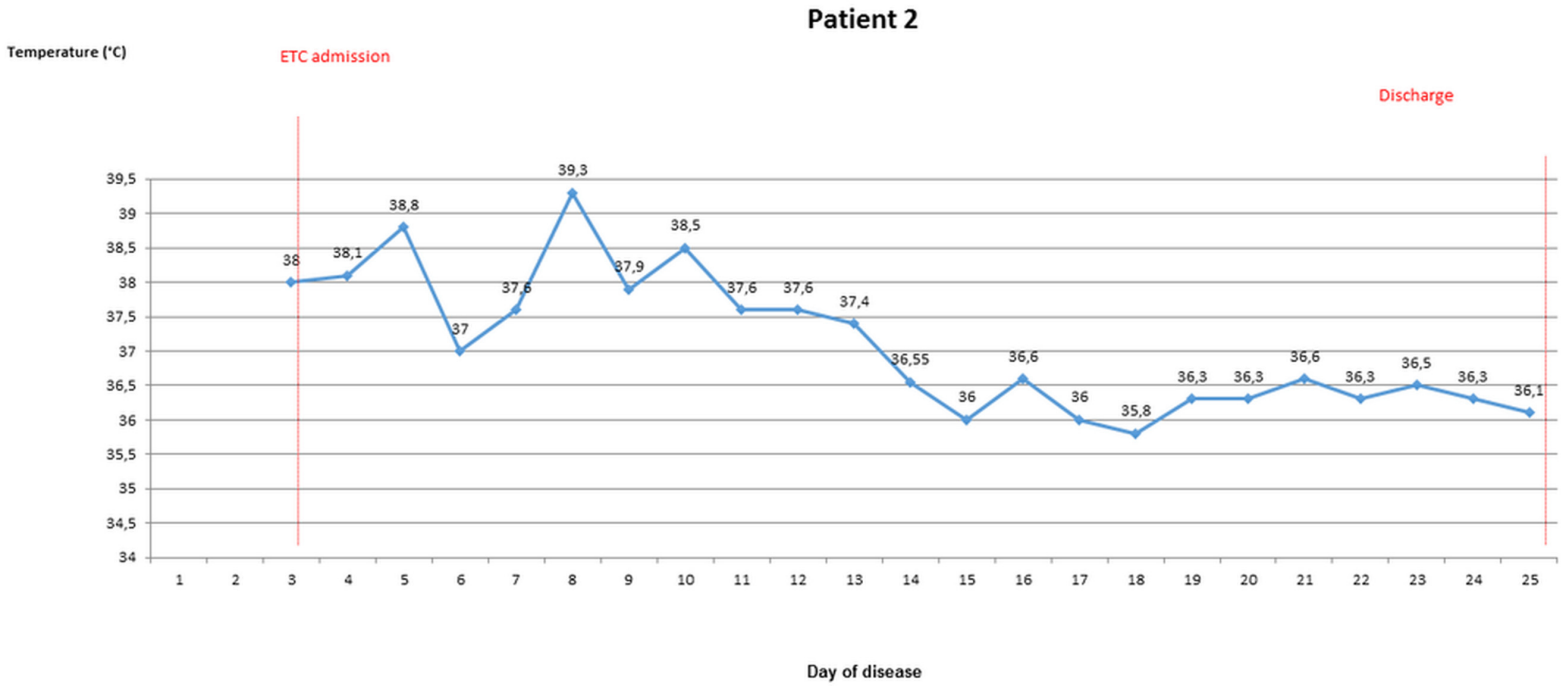
Evolvement of body temperature in patients treated in the Ebola Treatment Centre (ETC). In five inpatients of the ETC body temperature could be followed up from the second or third day of disease onwards. Four of those patients had fever at least on one day. Three patients showed a pattern with fever during the beginning of disease, which lasted no longer than until the 12^th^ day of disease. Three patients had high fever ≥ 38.5°C.

**Fig 5:**
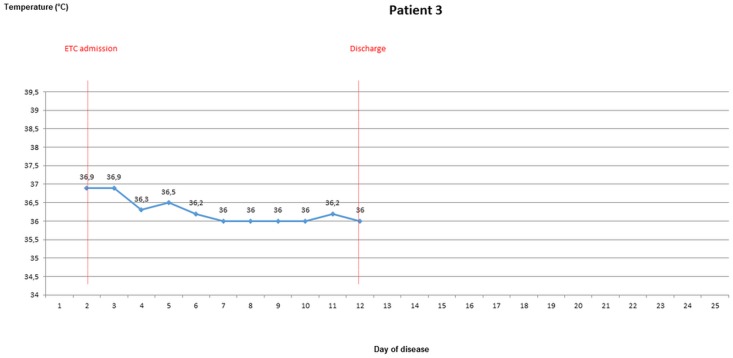
Evolvement of body temperature in patients treated in the Ebola Treatment Centre (ETC). In five inpatients of the ETC body temperature could be followed up from the second or third day of disease onwards. Four of those patients had fever at least on one day. Three patients showed a pattern with fever during the beginning of disease, which lasted no longer than until the 12^th^ day of disease. Three patients had high fever ≥ 38.5°C.

**Fig 6:**
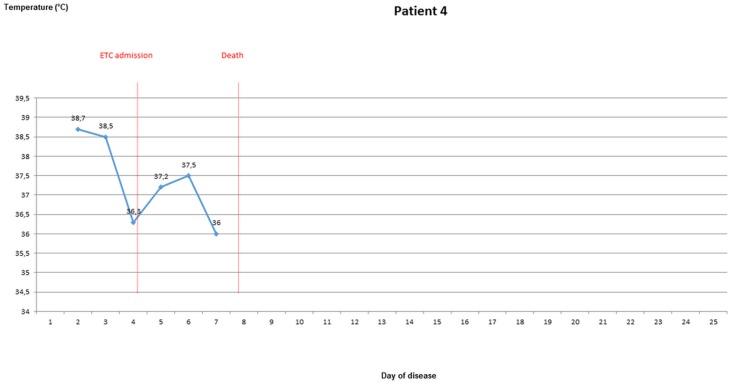
Evolvement of body temperature in patients treated in the Ebola Treatment Centre (ETC). In five inpatients of the ETC body temperature could be followed up from the second or third day of disease onwards. Four of those patients had fever at least on one day. Three patients showed a pattern with fever during the beginning of disease, which lasted no longer than until the 12^th^ day of disease. Three patients had high fever ≥ 38.5°C.

**Fig 7:**
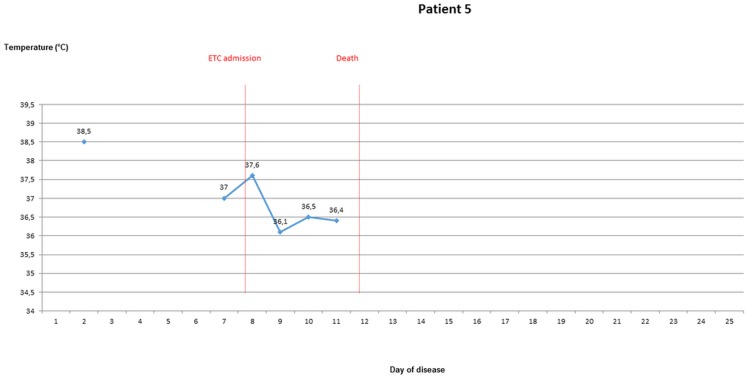
Evolvement of body temperature in patients treated in the Ebola Treatment Centre (ETC). In five inpatients of the ETC body temperature could be followed up from the second or third day of disease onwards. Four of those patients had fever at least on one day. Three patients showed a pattern with fever during the beginning of disease, which lasted no longer than until the 12^th^ day of disease. Three patients had high fever ≥ 38.5°C.

During the stay in the ETC, axillary temperatures of greater than 37.5° were measured in 10 out of 18 patients (55.6%), of greater than 38° in seven patients (38.9%). Temperature of ETC inpatients was taken and recorded 1.6 times per day (range 0.5–2.4) on average. For five ETC patients, body temperature was measured from the 3rd day of disease latest until discharge or death. Four of these were febrile on most days until day 10 (Figs 3–7).

### Self-reported symptoms of patients upon ETC admission

Prior to laboratory confirmation of BDBV infection, self-reported symptoms in 17 patients upon admission were asthenia (94.1%), fever (88.2%), anorexia (70.6%), joint and muscle pain (70.6%) and gastrointestinal manifestations (nausea, diarrhoea, vomiting (64,7%)). Less common self-reported symptoms included hiccups (11.8%) and haemorrhage (29.4%). (Tables 5 and 6)

**Table 5:** Signs and Symptoms developed by patients hospitalized in the Ebola Treatment Centre (ETC).

Sign/Symptom	Self-reported symptoms at admission (present/Total)	Signs/Symptoms assessed during hospital stay (present/Total)
Fever > 37.5°C (axillary)	15/17* (88.2%)	10/18 (55.6%)
Fever > 38.0°C(axillary)	4/17 (23.5%)	7/18 (38.9%)
Headache	7/17 (41.1%)	8/17 (47.1%)
Asthenia	16/17 (94.1%)	14/17 (82.4%)
Myalgia	12/17 (70.6%)*	12/17 (70.6%)
Arthralgia	*Joint or muscle pain	13/17 (76.5%)
Hiccups	2/17 (11.8%)	6/17 (35.3%)
Anorexia	12/17 (70.6%)	14/17 (82.4%)
Nausea	10/17 (58.8%)*	12/17 (70.6%)
Vomiting	* Nausea or Vomiting	11/17 (64.7%)
Sore throat / difficulty swallowing	3/17 (17.7%)	12/17 (70.6%)
Stomach pain	10/17 (58.8%)	11/17 (64.7%)
Tender abdomen	No data	10/17 (58.8%)
RUQ pain	No data	5/17 (29.4%)
Diarrhoea	10/17 (58.8%)	11/17 (64.7%)
Anuria	No data	1/17 (5.9%)
Dyspnoea	3/17 (17.7%)	4/17 (23.5%)
Cough	2/17 (11.8%)	8/17 (47.1%)
Chest pain	1/17 (5.9%)	5/17 (29.4%)
Back pain	No data	8/17 (47.1%)
Jaundice	1/17 (5.9%)	3/17 (17.7%)
Non—haemorrhagic rash	0/17 (0.0%)	1/17 (5.9%)
Hepatomegaly	No data	3/17 (17.7%)
Splenomegaly	No data	1/17 (5.9%)
Dehydration	No data	7/17 (41.1%)
Disorientation	No data	0/17 (0.0%)
Red or injected eyes	2/17 (11.8%)	2/17 (11.8%)
Epistaxis	1/17 (5.9%)	1/17 (5.9%)
Gingival/oral bleeding	1/17 (5.9%)	3/17 (17.7%)
Haemoptysis	No data	0/17 (0.0%)
Hematemesis	6/17 (35.3%)	2/17(11.8%)
Bloody stools	1/17 (5.9%)	1/17(5.9%)
Haematuria	1/17 (5.9%)	0/17 (0.0%)
Non-menstrual vaginal bleeding	3/17 (17.7%)	1/17 (5.9%)
Bleeding from injection site	0/17 (0.0%)	1/17 (5.9%)
Petechiae or cutaneous bleeding	0/17 (0.0%)	0/17 (5.9%)

*N<18 patients when data were missing.

**Table 6:** Syndromes in patients hospitalized in the Ebola Treatment Centre (n = 18).

Syndrome	Upon case notification = history of syndrome (yes/total)	TOTAL = At admission and during entire hospital stay (yes/total)	Proportion of patients who received adequate treatment^4^ for syndrome during entire hospital stay
**Haemorrhage** ^**1**^	5/17 (29.4%)	5/17 (29.4%)	Not applicable
**Gastrointestinal** ^**2**^	11/17 (64.7%)	13/17 (76.4%)	8/13 (61.5%)
**Pain** ^**3**^	15/17 (88.2%)	16/17 (94.1%)	14/16 (87.5%)

1: Haemorrhage: Any haemorrhagic sign, red/injected eyes not included.

2: Gastrointestinal: Nausea, diarrhoea, vomiting.

3: Pain: Headache, myalgia, arthralgia, back pain, chest pain, abdominal pain, sore throat, retro orbital pain.

4: Adequate Treatment: Oral or i.V. rehydration for gastrointestinal syndrome, analgesics for pain.

“Not applicable” for haemorrhage as blood transfusions were not carried out in the Ebola Treatment Facility and medication like Prothrombin Complex Concentrate was not available.

### Signs and Symptoms developed by 18 patients in the ETC

The majority (61%) of 18 patients reported to a hospital facility within the first three days of disease onset. The most frequently observed symptoms that were observed at some point during hospitalisation in the ETC were asthenia (82.4%), anorexia (82.4%), myalgia (70.6%), sore throat/difficulty swallowing (70.6%), arthralgia (76.5%) and nausea (70.6%). Fever as a sign was less common (55.6%). Hiccups were much more frequent during hospital stay (35.3%) than self-reported upon admission (Table 5). Gastrointestinal (GI) signs and symptoms were frequent (76.4%, Table 6). Ninety-four per cents of patients reported pain upon admission or during hospital stay. Haemorrhagic signs were much less prevalent (29.4%). One patient without history of mental disorder prior to admission showed disorientation and agitation upon discharge, which continued for at least one week thereafter. A longer follow-up period was not possible. The CFR in patients with gastrointestinal signs and symptoms was 53.8% vs. 25% in those without (p = 0.58), in patients with haemorrhage 60.0% vs. 41.7% in those without (p = 0.62).

### Concomitant malaria

Bioline rapid diagnostic tests (RDT) for malaria were performed on admission for nine of the 18 EVD confirmed ETC patients. Three RDT positive patients received an Artesunate combination therapy, together with a fourth patient who was treated presumptively. The nine cases where an RDT was not performed on admission included two where it was not applicable (one new-born, one patient who died immediately after admission), and seven for which it was not performed for unknown reasons. During the further hospital stay, RDTs were not performed. Concomitant malaria, as defined by a positive RDT or presumptive treatment (n = 4), did not influence the outcome (CFR 50% in patients with or without malaria).

### Time from sign or symptom onset until admission to hospital (Ebola Treatment Centre or other)

(Fig 8)

**Fig 8:**
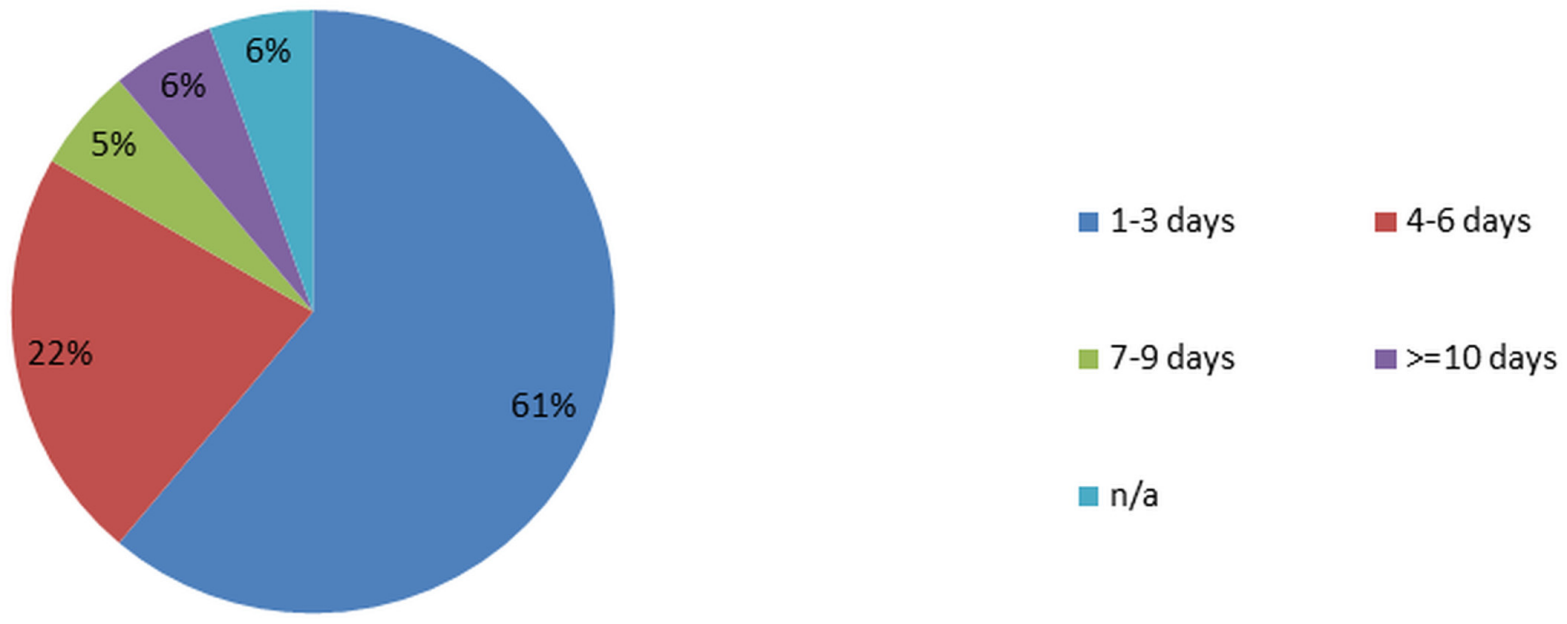
Time from sign or symptom onset until admission to hospital (Ebola Treatment Centre or other). 61% of the n = 18 confirmed EVD cases who were hospitalized in the ETC later showed up to a healthcare facility on their first until third day of disease. 22% of the cases did so between the 4^th^ and 6^th^ day, 5% from the 7^th^ until 9^th^ day, and 6 cases on the 10^th^ day of disease or beyond. One case was not applicable (n/a), because it was a baby born in the ETC.

### Incubation period and duration of EVD caused by BDBV

Based on information in the notification charts, the incubation period could only be determined for three ETC patients, and amounted to two, five and 27 days. The median duration of disease for the surviving ETC patients was 18 days (min. 8 days, max. 26 days), and 15 days (min. 4 days, max. 28 days) for those who died.

### Clinical course of confirmed BDBV infection in particular patients

**Fig 9:**
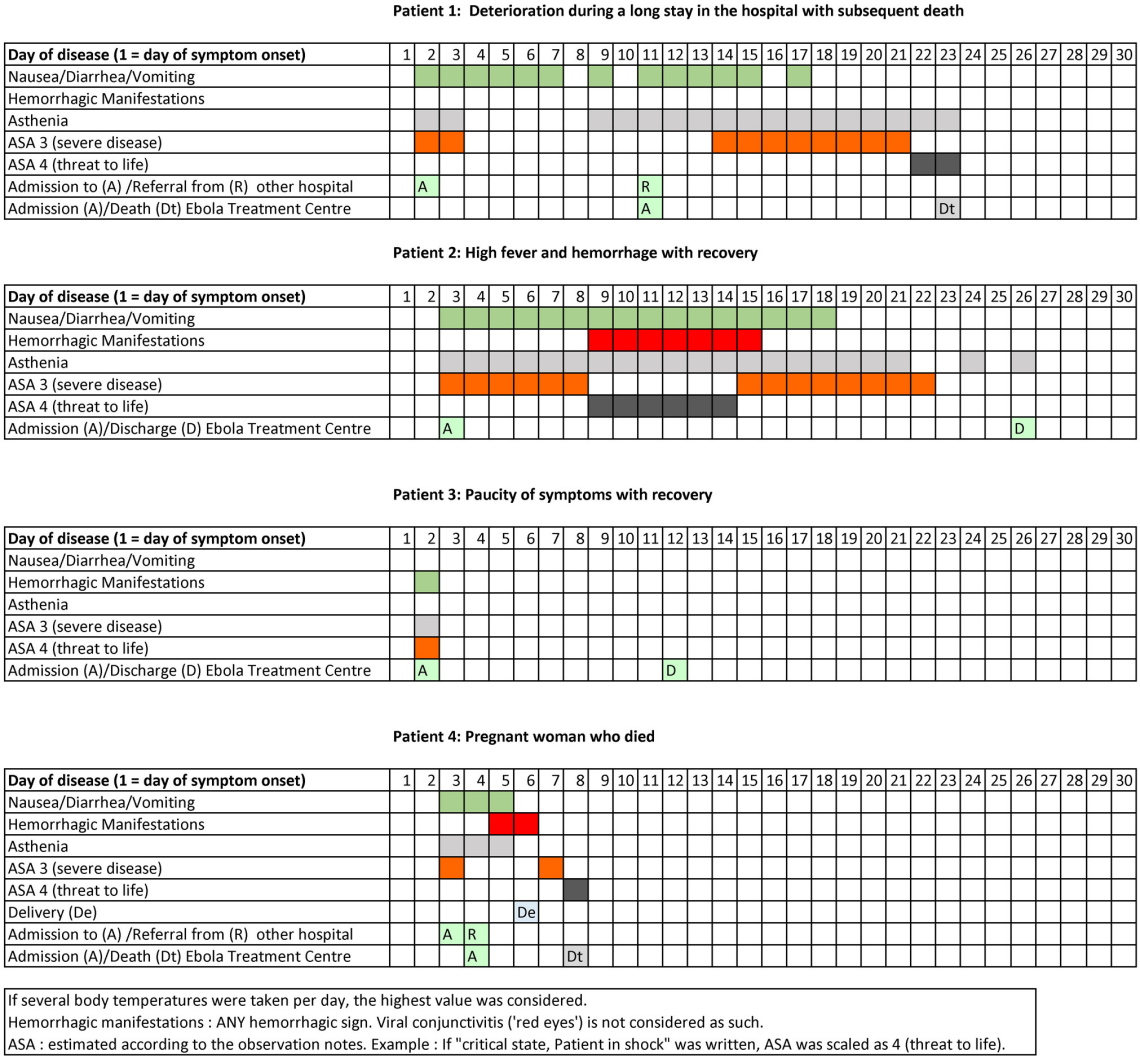
Evolvement of EVD of particularly informative inpatients in the Ebola Treatment Centre (ETC). The figure shows the evolvement of disease of two patients who succumbed due to EVD and two patients who survived it. All four patients are identical to patients No. 1 to 4 in Figs 3–7. The presence or absence of fever, gastrointestinal signs or symptoms, haemorrhagic signs, asthenia and a bad state of health (severe disease, threat to life) were documented on a daily basis, as well as admission to and discharge from the ETC.

The following case series provides details on the variable clinical course of BDBV disease (Fig 9).

The patients 1 to 4 mentioned are identical with patients 1 to 4 from Figs 3–7 (body temperature evolvement).

#### Patient 1

A 27-year-old female, admitted to a regular hospital on the second day of disease with dysphagia, gastrointestinal manifestations and a body temperature of 37°C. She did not appear to be very severely ill at the beginning. From day two until day eight of disease, there were only short remarks in the ward rounds documenting gastrointestinal manifestations. Body temperature was only recorded with gaps and from the third day of disease onwards. Admitted as a suspect case to the ETC on day nine. Laboratory confirmation of EVD took place on day eleven; she was subsequently transferred to the confirmed ward of the ETC. Dehydration signs with persistent skin pinch were documented around day 14; ORS and intravenous fluids were administered. Afterwards slight improvement, patient tried to flee the isolation ward on day 15. On day 20, the patient deteriorated clinically once again with hypothermia (35.2°C) and asthenia. On day 22, she revealed fever (38.4°C), crackles audible without a stethoscope and tachypnea. On day 23, she died.

#### Patient 2

A 32-year-old female, who was admitted to the ETC on her third day of disease. Standard combination treatment, as described in the Medical Treatment Section below, was implemented immediately. A long duration (26 days) of disease, including a period of six days when the patient was in a life-threatening condition, was observed. During the first half of the hospital stay she was febrile, with risk of dehydration most of the time, and with haemorrhagic manifestations (hematemesis, epistaxis, gingival bleeding). At day 11 of disease, a poor prognosis was stated. Nursing with intensified oral fluid support was carried out subsequently and enhanced from day 20 onwards with intravenous fluid support. At day 22 of disease, improvement of the clinical condition, “the patient ate a sugar cane“. She finally recovered without sequelae.

#### Patient 3

A 60 year old male whose symptoms reported in Fig 9 were present only on the day of hospital admission (second day of disease). Apart from this only “cough” was recorded on the third day of disease. There was a short duration of illness (3 days) with eventual recovery. Paucity of symptoms is reliable as ward rounds were documented in the patient file and the absence of symptoms was confirmed.

#### Patient 4

A 29-year-old female, 7 months pregnant on admission. On the fourth day of disease, she was admitted to the ETC and presented fever, vomiting, dysphagia and diarrhoea. A 50% cervical effacement with dilation at 4cm was stated. She was confirmed to suffer from EVD by PCR the same day. Dystocia was stated at the end of day five, with 100% cervical effacement and dilation at 8 cm. Non—assisted delivery took place on the sixth day of disease, when ten International Units (IU) of Oxytocine were administered intramuscular (i.m.). Intravenous fluids, Methergine, Cefixime, Iron/Folic Acid and Plumpynut were given post partum. On the day after delivery, the patient deteriorated rapidly, presenting drowsiness and wheezing audible without a stethoscope. Antibiotherapy was consecutively switched from Cefixime to Ceftriaxone. Dyspnoea, coma stage 1b, light exophthalmos, cold limbs and sub icterus were stated on the second day post partum. The patient died the same day. The child was born with an APGAR score of 8/10/10, remained asymptomatic until day four, became febrile (38.5°C) on day five, deteriorated and died on the eighth day after birth.

### Monitoring of patients

Body temperature was documented on at least 80% of days of hospitalisation in 14, signs and symptoms in 15, and both body temperature as well as signs and symptoms in 13 out of 18 ETC patients. CFR was slightly, but insignificantly lower in patients for whom body temperature and signs/symptoms were thoroughly observed and documented (CFR 46.2% vs. 60.0%; p = 1). Blood pressure was not taken due to biosafety considerations.

### Medical treatment in the ETC

Ten out of the 18 ETC patients (55.6%) received an oral standard combination treatment of Cefixime, multivitamin and paracetamol. These patients had an insignificantly lower CFR (40.0% vs. 62.5%, p = 0.64). To prevent or treat dehydration, ORS (38.9%) and I.V. fluids (27.8%) were administered, but only 61.5% of patients suffering from gastrointestinal manifestations including nausea received oral or I.V. rehydration. Patients receiving oral rehydration solution or I.V. fluids had an insignificantly lower CFR (37.5% vs. 60.0%, p = 0.64). Analgesics including paracetamol and Tramadol were administered and recorded in 77.8% of cases. All four patients (22.2%) who were diagnosed with malaria received artesunate-based antimalarial combination therapy, which did not have a detectable effect on the CFR. Half of the patients received some medication intravenously, while blood transfusions were not carried out.

### Quality of documentation

Fever was indicated as ‘yes’ upon case notification for 15 out of 17 patients who were later admitted to the ETC. It remains unclear whether this means that the patient had fever at the time of notification or reportedly the days before. Only in six out of those 17 cases axillary body temperature was taken and recorded on admission by the ETC team, and fever was confirmed in four of them (66.7%). During the first day in the ETC, fever was confirmed in only three out of 18 patients. Medication was not systematically recorded in the patient files.

The proportion of clinical records with a mismatch between the original established inside and the copy established outside HRZ from memory concerning signs and symptoms ranged from 22.6% for physical asthenia to 30.4% for petechiae. On average, the proportion of records with a mismatch was 27.3% across signs and symptoms. Doctors’ notes on the original were sometimes more, sometimes less elaborate than on the copy depending on workload and availability of information in the HRZ. Copies were better readable than the originals, which were often bleached by chlorine spills and spraying. In patient files from healthcare facilities other than the ETC, there were significant gaps in the documentation of signs, symptoms, vital parameters and treatment.

## Discussion

The second known outbreak of EVD cause by BDBV produced additional detailed clinical data from patients hospitalized in the ETC. A limitation was the small sample size, limiting the power of our study to identify modest differences as significant. Another limitation was the partially poor quality of clinical documentation.

The method used for data transfer from inside to outside the ETC’s HRZ proved to be unreliable and needs to be improved: for clinical documentation one should not rely on health workers’ memory. We discourage this practice for future outbreaks. Towards the end of the outbreak contaminated patient files located inside the HRZ were photographed with a high-resolution digital camera from a safe distance of two metres across the HRZ barrier. This method of transferring clinical data from the inside to the outside of the HRZ without loss or alteration of data was quick, easy, and safe, did not require special equipment, and allowed immediate verification of the soft copy.

With 53.8% overall CFR was similar to the one reported from the first known EVD outbreak caused by BDBV in Uganda 2007 (40%, [4]). CFR of confirmed ETC cases (50.0%) is likely a relatively valid estimate, but could be somewhat overestimated if severe cases were overrepresented among the cases (self-) referred to the ETC. CFR of community probable cases (93.8%) is likely overestimated because of selection bias in favour of fatal cases fulfilling the case definition who could not be tested before death, whereas the CFR of community confirmed cases (22.2%) is likely an underestimate by selection bias in favour of individuals who survived without hospital treatment and were identified as EVD cases during convalescence by serology (IgM). Hence aggregated data on community CFR would only be accurate if proportions [detected community probable cases/total community probable cases] and [detected community confirmed cases/total community confirmed cases] would be equal.

There was an uncertainty about the definition of fever upon case notification: In reality, current fever *and* the history of fever were taken registered as ‘fever’ when notifying suspect cases. Even though the mere history of fever was not a part of the MOH case definition, it was considered because of the possibility that the patient was not febrile any more when presenting at the ETC, e.g. after taking antipyretics. Nausea and vomiting should be documented separately, as only the latter can cause dehydration, which is a frequent complication of EVD and may be associated with a poor prognosis.

The increased morbidity and mortality of EVD ETC cases in females and in ages 15–55 could be attributed to the increased exposure of those groups to the diseased or dead bodies entailing an increased viral exposure, as discussed in the press in the context of the 2014 West African Ebola outbreak. [17]

Gastrointestinal signs with the potential for dehydration were very frequent, underlining the importance of good fluid monitoring and management. Thorough monitoring of patients was associated with an insignificantly higher survival rate. Monitoring should be improved further by introducing the monitoring of fluid management with input/output charts, which may prevent dehydration in patients suffering from vomiting or diarrhoea. Monitoring blood pressure should be introduced among confirmed patients as well, since it could help in the prevention and early detection of shock. Stethoscopes, which are normally used for taking the blood pressure, could compromise biosafety e.g. by punching holes into the hoods that are now a standard component of PPE used for EVD. However, healthcare workers could either take only the systolic blood pressure by palpation, or use an electronic sphygmomanometer. Cuffs used on confirmed patients exclusively could be decontaminated by wiping disinfection, since there is no risk of cross-contamination between confirmed EVD patients. The association between an orally administered systematic treatment and a decreased CFR is, apart from being insignificant, susceptible to confounding, as patients in a poor state of health might be unable to take it.

Pain management could be improved by administering targeted analgesic treatment according to the WHO scheme for cancer pain relief [18]. Antibiotic therapy could be systematic with Cefixime for all patients to cover co-infections like typhoid fever, and targeted when another bacterial co-infection is suspected. For malaria we recommend presumptive treatment for all patients with artesunate combination therapy, since an RDT alone is not sufficiently reliable and handling an RDT is an additional biohazard. [19]

Fever showed up in patients only intermittently and not always upon admission, making it thus an unreliable major criterion for the case definition. Paracetamol is widely available in DRC, thus potentially ‘hiding’ fever and thus rendering a case definition based on ‘fever’ problematic. It remained unclear to what extent the absence of fever in some patients was due to undocumented paracetamol consumption prior to and after hospital admission. The use of antipyretics should thus be documented in the notification chart. There were high prevalence of asthenia and anorexia upon admission, but those symptoms are unspecific and often occur in common tropical diseases, e.g. malaria. Sore throat, another frequent symptom in EVD, may be more specific, as it does not occur in diseases typically to be considered as differential diagnosis. The more frequent occurrence of hiccups during hospital stay than upon admission is in line with them being a late sign [13]. Bleeding was uncommon. The differential diagnoses stated in cases, which later turned out to be BDBV infection instead, underline the high prevalence of non-specific symptoms. This, together with the non-specificity of other symptoms, underlines the importance of swift laboratory confirmation of suspect cases by an on-site laboratory. A calculation of an incubation period of 11.3 days based on only 3 patient might not be representative, but coincides with the one calculated by the WHO response team for the 2013 Western Africa EVD epidemic due to EBOV infection (11.4 days). The incubation period of 27 days observed in one EVD patient might be an outlier beyond the regular maximum incubation period of 21 days, as also seen in 5% of the EVD patients evaluated by the WHO response team in West Africa. [1]

To conclude, EVD caused by BDBV is rare but challenging due to the little knowledge we have about it. More research in terms of symptom prevalence, treatment and outcome is needed.

## Supporting Information

S1 Text Case definition of EVD. Case definition by Congolese Ministry of Health used in Isiro, DRC.
(PDF)

S1 Table Notification Chart. Notification chart used in Isiro, DRC.
(PDF)

S2 Table Symptom Follow up Chart. Symptom Follow up Chart used in Isiro, DRC.
(PDF)
