# Supplementary material for: Ebola Virus Disease Outbreak in Isiro, Democratic Republic of the Congo, 2012: Signs and Symptoms, Management and Outcomes
Source: PLoS One. 2015 Jun 24;10(6):e0129333. doi: 10.1371/journal.pone.0129333 (PMC4479598; doi:10.1371/journal.pone.0129333)
Supplement: S1 Text — Case definition by Congolese Ministry of Health used in Isiro, DRC. (PDF) [file pone.0129333.s001.pdf]

1.0 Case Definition

CASE DEFINITION OF EBOLA HAEMORRHAGIC FEVER
Applies to the Haut Uele and Bas Uele Districts
DURING THE EPIDEMIC PERIOD from 1st of MAY 2012 ONWARDS

To be used by community health workers
ALERT CASE
Any person with a sudden onset of high fever
OR: Bleeding or red coloured stools or blood in urines
OR: Sudden death

IF an alert case (dead or alive) is identified:
Notify to a an Outreach Team or to the closest Health Centre

To be used by Outreach Teams or Health Centres
SUSPECT CASE:
- Any person, dead or alive who shows or did show a clinical presentation of fever[1] with haemorrhagic signs/ bleeding
- OR any person, dead or alive who shows or shows or did show a clinical presentation of fever with at least three of the following symptoms :
  - Headache
  - Anorexia / Loss of appetite
  - Intense fatigue
  - Muscle or joint pain
  - Difficulties to breathe
  - Vomiting
  - Diarrhoea
  - Abdominal pain
  - Difficulties to swallow
  - Hiccups

- **OR** any person, dead or alive who shows or did show a clinical presentation of fever together with a notion with a probable or confirmed case of haemorrhagic fever.
- **OR** Any unexplained death (with a clinical history of suspicion)

When a suspect case is identified
If the subject is alive:
1. The subject should be explained that she/he has to be sent to a hospital in order to receive appropriate Medical care
2. One should report the case to the coordination team in order to transport the patient
3. One should fill in the contact list

If the subject is dead:
1. One should report the death to the coordination team in order to facilitate the dead body management
2. One should fill in the contact list

To be used at hospital level and by the surveillance team

PROBABLE CASE : Any person corresponding to the definition of a suspect case who, additionally, had a contact with a probable or confirmed case during three weeks preceding the onset of symptoms

CONFIRMED CASE: Suspect or probable case with a positive laboratory result (PCR or Serology)

* Contact: Any person who was a contact with case (within the last 21 days incubation period) according to at least one of the following modalities:
1. Has slept in the same household as the case within the month preceding the onset of symptoms
2. Has had a direct physical contact with the case (dead or alive) during his/her disease
3. Has touched the body fluids of a person during his/her disease
4. Has manipulated the clothes or laundry of a patient
5. Has been breastfed

[1] Definition of fever: >=37.5°C axillary
