# Supplementary material for: Ebola Virus Disease Outbreak in Isiro, Democratic Republic of the Congo, 2012: Signs and Symptoms, Management and Outcomes
Source: PLoS One. 2015 Jun 24;10(6):e0129333. doi: 10.1371/journal.pone.0129333 (PMC4479598; doi:10.1371/journal.pone.0129333)
Supplement: S1 Table — Notification chart used in Isiro, DRC. (PDF) [file pone.0129333.s002.pdf]

Fiche de Notification de Fièvre Hémorragique Virale (EBOLA)

Numéro d'identification du malade (ISIRO)

Date de notification: ____/____/_____ (J, M, A)

Fiche de Notification Remplie par:

Nom: ______________________ Position: ______________________
Zone de Santé: ______________________ District Sanitaire:______________________
Téléphone: ______________________ E-mail:______________________

Section 1. Information sur le Patient

Nom de famille: ______________________ Autres Noms:______________________
Date de Naissance: ____/____/______ (J, M, A) **OU:** Age: _______ ☐ Années ☐ Mois Sexe: ☐ Masculin ☐ Feminin
Numéro de téléphone: ______________________ Téléphone d'un membre de la famille: ______________________

Lieu de résidence permanente:

Nom du Chef de famille: ______________________ Village/Ville: ______________________
Aire de Santé: ______________________ Zone de Santé:______________________
District Sanitaire: ______________________ Nationalité:______________________
Pays de Résidence: ______________________ Groupe Ethnique: ______________________

Occupation:

☐ Planteur/Eleveur ☐ Boucher ☐ Chasseur/Vendeur de viande de brousse ☐ Mineur ☐ Ménagère ☐ Etudiant ☐ Enfant
☐ Personnel de Santé: Activité: ______________________ Centre de Santé: ______________________
☐ Autre, précisez le métier: ______________________

Endroit où le patient est tombé malade:

Village: ______________________ Zone de Santé: ______________________ District Sanitaire: ______________________

Section 2. Signes Cliniques et Symptômes

Date de début des symptômes: ____/____/______ (J / M / A)

Fièvre: ☐ Oui ☐ Non ☐ Inconnu Date de début de la Fièvre: ____/____/______ (J/M/A)
Si Oui, temp.: _________º C Mesure: ☐ Creux axillaire ☐ Bouche ☐ Rectale

Notez les signes et symptômes observés ou ressentis entre la date de début et la date du prélèvement:

Signes et symptômes cliniques: (cochez tous les signes et symptômes présents)

Nausées/Vomissements	☐ Oui ☐ Non ☐ Inc
Diarrhées	☐ Oui ☐ Non ☐ Inc
Douleurs musculaires/articulaires	☐ Oui ☐ Non ☐ Inc
Fatigue intense	☐ Oui ☐ Non ☐ Inc
Douleurs abdominales	☐ Oui ☐ Non ☐ Inc
Céphalées	☐ Oui ☐ Non ☐ Inc
Conjunctivite	☐ Oui ☐ Non ☐ Inc
Eruptions cutanées	☐ Oui ☐ Non ☐ Inc
Mal à la gorge	☐ Oui ☐ Non ☐ Inc
Toux	☐ Oui ☐ Non ☐ Inc
Difficultés à avaler	☐ Oui ☐ Non ☐ Inc
Difficultés à respirer	☐ Oui ☐ Non ☐ Inc
Hoquet	☐ Oui ☐ Non ☐ Inc
Perte d'appétit, anoréxie	☐ Oui ☐ Non ☐ Inc

Autres signes ou symptômes cliniques ☐ Oui ☐ Non ☐ Inc
If Oui, précisez: ______________________

Signes et symptômes hémorragiques: (cochez tous les signes et symptômes présents)

Saignements inexpliqués	☐ Oui ☐ Non ☐ Inc
**Si Oui:**	
Saignements des gencives	☐ Oui ☐ Non ☐ Inc
Saignements aux sites d'injections	☐ Oui ☐ Non ☐ Inc
Saignements vaginaux en dehors des règles	☐ Oui ☐ Non ☐ Inc
Saignements du nez / Epistaxis	☐ Oui ☐ Non ☐ Inc
Petechies / Purpura	☐ Oui ☐ Non ☐ Inc
Conjunctivite hémorragique	☐ Oui ☐ Non ☐ Inc
Sang dans les selles ou Méléna	☐ Oui ☐ Non ☐ Inc
Hématémèse / Vomissements sanglants	☐ Oui ☐ Non ☐ Inc
Hématurie (sang dans les urines)	☐ Oui ☐ Non ☐ Inc
CIVD / Shock	☐ Oui ☐ Non ☐ Inc
Autres signes hémorragiques	☐ Oui ☐ Non ☐ Inc

Si Oui, précisez: ______________________

Autres signes and symptômes spécifiques: (cochez tous les signes et symptômes présents)

Signes neurologiques	☐ Oui ☐ Non ☐ Inc	Pharyngite	☐ Oui ☐ Non ☐ Inc
Douleurs Retro-orbitales	☐ Oui ☐ Non ☐ Inc	Douleurs rétrosternales	☐ Oui ☐ Non ☐ Inc
Photophobies	☐ Oui ☐ Non ☐ Inc	Ulcérations de la muqueuse buccale	☐ Oui ☐ Non ☐ Inc
Jaunisse	☐ Oui ☐ Non ☐ Inc	Pharyngite à membranes	☐ Oui ☐ Non ☐ Inc
Encéphalite	☐ Oui ☐ Non ☐ Inc	Adénopathies (ganglions) cervicales	☐ Oui ☐ Non ☐ Inc
Rétinite	☐ Oui ☐ Non ☐ Inc	Proteinurie	☐ Oui ☐ Non ☐ Inc

Notez la liste d'éventuels autres signes cliniques charactéristiques:
____

Fiche de Notification de Fièvre Hémorragique Virale (EBOLA)

Numéro d'identification du malade (ISIRO)

Section 3. Patient/Clinique

Notification du cas par: (cochez tous les choix possibles)
☐ Surveillance Passive ☐ Surveillance Active ☐ Dans la Communauté ☐ Hôpital ☐ Décès ☐ Rumeurs
☐ Autre, précisez: ____

Etat du malade lors de la détection ou l'hospitalisation: ☐ Vivant ☐ Décédé

Hospitalisation:

Est-ce que le malade a été hospitalisé? ☐ Oui ☐ Non **Si Oui, continuez à remplir:**

Date d'hospitalisation: ____/____/______ (J, M, A) Centre de Santé: ____________________ Zone de Santé: __________________

Est-ce que le malade a été isolé à l'admission? ☐ Oui ☐ Non ☐ Inc Malade actuellement isolé? ☐ Oui ☐ Non ☐ Inc

Date de sortie de l'hôpital: ____/____/______ (J, M, A)

Etat actuel du malade: ☐ Vivant ☐ Décédé Si décédé, precisez la date de la mort: ____/____/______ (J, M, A)

Lieu du décès: ☐ Communauté ☐ Hôpital ☐ Autre: ________________________________

Village: ______________________ Zone de santé: ______________________ District sanitaire: ________________________

Lieu de l'enterrement: Village: _____________________ Zone: _____________________ District: _______________________

Section 4. Epedemiologie/Facteurs d'Expositions

Dans les **3 semaines** précèdant le début des symptômes:

1. Est-ce que le malade a eu un contact direct ou a été exposé aux animaux suivants?
☐ Chauve-souris ☐ Singes ☐ Rongeurs ☐ Bétail ☐ Oiseaux ☐ Autres: ____________________________________
Lieu: _________________________________ Date d'exposition: ____/____/______ (J, M, A)

2. Est-ce que le malade a assisté à des funérailles? ☐ Oui ☐ Non ☐ Inc Date: ___/___/____ - ___/___/____ (J, M, A)
Si oui, est-ce que le malade a participé aux funérailles? ☐ Oui ☐ Non ☐ Inc
Nom de la personne décédée:_______________________________________ Lieu:_________________________________

3. Est-ce que le malade a eu des contacts avec un cas d'Ebola (confirmé ou suspect)? ☐ Oui ☐ Non ☐ Inc
Si oui, à quel endroit?: _______________________________ Date(s) de contact: ___/___/____ - ___/___/____ (J, M, A)
Nom de la personne: _______________________________ Pendant les contacts, le suspect était: ☐ Vivant ☐ Mort ☐ Inc

4. Est-ce que le malade a consulté un médecin traditionnel pour sa maladie? ☐ Oui ☐ Non ☐ Inc
Si oui, précisez où: __________________ et quand: ___/___/____ (J, M, A) A-t-il reçu un traitement? ☐ Oui ☐ Non ☐ Inc

5. Est-ce-que le malade a voyagé en dehors de sa maison ou de son village <u>avant</u> d'être malade? ☐ Oui ☐ Non ☐ Inc
Si oui, précisez où: ________________________________ Date: ___/___/____ - ___/___/____ (J, M, A)

Est-ce que le malade a voyagé en dehors de son village <u>durant</u> la maladie? ☐ Oui ☐ Non ☐ Inc
Si oui, précisez le village: _______________________________ ou le Centre de Santé: ______________________________

Section 5. Prélèvements Biologiques pour le Laboratoire

- Chaque prélèvement doit être correctement identifié: **Nom du patient, numéro d'identification**, **date de prélèvement** et **type de prélèvemen**t.
- Les prélèvements doivent être envoyé réfrigérés ou congelés avec un emballage correct pour la sécurité.
- Volume demandé**: <u>4ml</u>** (volume minimum: **2ml**)

Sang complet:

1. Prélevé à l'admission sur tube EDTA (bouchon pourpre), tube sec (bouchon rouge) ou tube hepariné (bouchon vert)
2. Sang cardiaque prélevé en post-mortem

<u>Prélèvement 1:</u>
Date du prélèvement: ____/____/______ (J, M, A)

Type de prélèvement:
☐ Sang complet
☐ Biopsie ou Autopsie, précisez l'organe prélevé: _______________________________
☐ Autre type de prélèvement, précisez: ______________________________________

<u>Prélèvement 2:</u>
Date du prélèvement: ____/____/______ (J, M, A)

Type de prélèvement:
☐ Sang complet
☐ Biopsie ou Autopsie, précisez l'organe prélevé: _______________________________
☐ Autre type de prélèvement, précisez: ______________________________________
