# Supplementary material for: Ebola Virus Disease Outbreak in Isiro, Democratic Republic of the Congo, 2012: Signs and Symptoms, Management and Outcomes
Source: PLoS One. 2015 Jun 24;10(6):e0129333. doi: 10.1371/journal.pone.0129333 (PMC4479598; doi:10.1371/journal.pone.0129333)
Supplement: S2 Table — Symptom Follow up Chart used in Isiro, DRC. (PDF) [file pone.0129333.s003.pdf]

[illegible]

**Family name:**	**DD / MM / YYYY**
**First name:**	**Onset of symptoms:** / /
**Identifier No.:**	**Date of admission:** / /
**Age:** **Sex:**	**Date of discharge:** / /

Adm = day of admission

**Day**	**Adm**	**2**	**3**	**4**	**5**	**6**	**7**	**8**	**9**	**10**	**11**	**12**	**13**	**14**
**Symptoms** (check all that apply)														
Headache														
Asthenia (severe weakness)														
Myalgia														
Arthralgia														
Hiccups														
Anorexia														
Nausea														
Vomiting														
Sore throat/ difficulty swallowing														
Stomach pain														
Tender abdomen														
RUQ pain														
Diarrhoea														
Anuria														
Dyspnoea														
Cough														
Chest pain														
Back pain														
Jaundice														
Non-haemorrhagic rash														
Hepatomegaly														
Splenomegaly														
Dehydration														
Disorientation														

**Haemorrhagic signs**														
Red or injected eyes														
Epistaxis														
Gingival/oral bleeding														
Haemoptysis														
Haematemesis														
Bloody stools														
Haematuria														
Non-menstrual vaginal bleeding														
Bleeding from injection site														
Petechiae or cutaneous bruising														

**Any other symptoms**														
